# In Vivo Effectiveness of *Pleurotus ostreatus* in Degradation of Toxic Metabolites of Filamentous Fungi Such as Aflatoxin B1 and Zearalenone

**DOI:** 10.3390/metabo15010020

**Published:** 2025-01-05

**Authors:** Agnieszka Zapaśnik, Marcin Bryła, Adrian Wojtczak, Barbara Sokołowska

**Affiliations:** 1 Department of Microbiology, Waclaw Dabrowski Institute of Agricultural and Food Biotechnology—State Research Institute, Rakowiecka 36, 02-532 Warsaw, Poland; adrian.wojtczak@ibprs.pl (A.W.); barbara.sokolowska@ibprs.pl (B.S.); 2 Department of Food Safety and Chemical Analysis, Waclaw Dabrowski Institute of Agricultural and Food Biotechnology—State Research Institute, Rakowiecka 36, 02-532 Warsaw, Poland; marcin.bryla@ibprs.pl

**Keywords:** secondary metabolites, enzymes, mycotoxins, detoxification

## Abstract

Background/Objectives: Mycotoxins, secondary metabolites synthesized by filamentous fungi, have been classified as dangerous substances and proven to be carcinogenic, as well as to have genotoxic, nephrotoxic, hepatotoxic, teratogenic, and mutagenic properties. Despite numerous trials to develop an effective and safe-for-human-health method of detoxification, there is still a high risk associated with the occurrence of these toxins in food and feed. Biological methods of food preservation are an alternative option to conventional chemical and physical methods, characterized by their less negative impact on human health as well as their high efficiency against filamentous fungi and other foodborne pathogens. Mycoremediation is a new biotechnique based on the capability of fungi to detoxify matrices from various pullulans. Ligninolytic enzymes produced by white rot fungi (WRF) characterize a high efficiency in the degradation of various mycotoxins. Methods: In our study, *Pleurotus ostreatus*, as a representative of WRF, was cultivated on a medium contaminated by AFB1 and ZEN (mushroom substrate and maize) in a few variants of concentration. After the cultivation, medium and fruiting bodies were collected and analyzed with the usage of HPLC and LC/MS methods. Results: The reduction oscillated between 53 and 87% (AFB1) and 73 and 97% (ZEN) depending on the initial concentration of toxins in the medium. Grown fruiting bodies contained insignificant amounts of both toxins. Conclusions: These findings confirm the potential of *P. ostreatus* as an effective biological agent for reducing mycotoxins in contaminated medium, highlighting its applicability in developing sustainable and safe methods for detoxification.

## 1. Introduction

Mycotoxins are secondary metabolites biosynthesized by filamentous fungi widespread in all kinds of food products, as well as feeds [1,2,3]. About 25% of the world’s food crops are polluted by mycotoxins, according to a statement of the FAO (Food and Agricultural Organization) in 1985 [4], but Escola et al. [5] provided a new estimation which suggests a variation between 60 and 70%, according to reports about detected mycotoxins in recent years.

Aflatoxins belong to the group of the most harmful mycotoxins to human health. This toxin may be found in soil as well as in food or feed, but the most common contaminations of aflatoxins, especially AFB1, occur in grains, nuts, spices, tea, and dairy products. The main producer of these toxins is *Aspergillus flavus*, but some other strains such as *Aspergillus parasiticus* [6,7] also produce them. According to the opinion of the IRAC (International Agency for Research on Cancer) [8], aflatoxin B1 (AFB1) is classified as a substance with a proven carcinogenic effect on mammals (Group 1) [9,10]. As a matter of fact, about 4.5 billion people worldwide, especially in developing countries, are presumedly exposed to AFB1 present in their daily diets, which may lead to serious health problems, including worsening liver and kidney conditions [6,11,12]. Aflatoxins are carcinogenic to fish, mice, rats, ducks, and monkeys, primarily inducing hepatocellular carcinoma and cholangia carcinoma in liver. Furthermore, AFB1 displays significant oral carcinogenicity in a wide range of animal species [7,13]. According to Wogan and Newberne [14], a dosage of about 0.2 μg/kg per day may stimulate carcinoma in rats. AFB1, given in a dose of about 1 mg/kg over 14 days, caused the deaths of two out of three dogs taken for this experiment [14]. These examples prove the high carcinogenicity of AFs for animals; but, taking into consideration the dependency between primary liver cancer and diet in humans, there is a high possibility that AFs are toxic for humans to the same degree as for other mammals [13]. Referring to the literature data, AFs display hepatotoxic, nephrotoxic, genotoxic, mutagenic, immunosuppressive, teratogenic, and cytotoxic effects [15,16].

Another mycotoxin with a negative impact on human health is zearalenone (ZEN). ZEN is biosynthesized by some *Fusarium* strains, such as *Fusarium culmorum* or *Fusarium graminearum*, and might be found predominantly in maize, but is also found in other grain crops, especially in temperate cultivation regions [17]. From a toxicological point of view, ZEN is not as harmful as AFB1, but has a significant impact on endocrine disruption. In the opinion of the IRAC, this toxin belongs to Group 3 due to the insufficient evidence of carcinogenic effects in human or animal bodies. ZEN represents xenoestrogens, the chemical structures of which are similar to natural estrogens. This similarity allows for binding to estrogenic receptor sites and amplified estrogenicity [18,19,20]. Low levels of progesterone and serum testosterone in the bloodstream during constant exposure to ZEN may lead to problems with fertility in animals, including cows, rats, and pigs. The contamination of these mycotoxins may occur during the different stages of food manufacturing, including cropping, harvesting, and storage [21,22].

Regarding the risk associated with mycotoxin contamination, there are a few methods of detoxication that work against it, including chemical (hydrolytic substances, oxidation), physical (heat, ionizing radiation, ultraviolet light), and biological (bacteria, yeast, enzymes) [23]. In recent years, the mycoremediation process has attracted the attention of scientists due to its high efficiency in neutralizing of food contaminants without incurring negative effects on the quality of food products or on human health [24]. Bioremediation by microorganisms has been shown to be a cheap method with high potency against different groups of food contaminants such as pesticides [25], polycyclic aromatic hydrocarbon (PAH) [26], heavy metals [27], and mycotoxins [28]. This biotechnique is based on the ability of microorganisms (or biomass) to reduce toxins by a number of different mechanisms, including biosorption, bioconversion, biodegradation, or bioaccumulation [29]. A relatively new approach is the biological degradation of mycotoxin by edible mushrooms, including genera such as *Pleurotus osteratus*, *Phanerochaete chrysosporium*, *Ganoderma lucidum*, *Trametes versicolor*, *Irpex lacteus, Lentinus edodes*, and *Coriolus versicolor* [30].

*Pleurotus* fungi are classified under the phylum Basidiomycota, order Agaricales, and family Pleurotaceae [31]. These mushrooms also represent the WRF (white rot fungi) group. Organisms belonging to WRF are characterized by their ability to grow on wood or trees, penetrating their cell cavities and producing ligninolytic enzymes such as laccase or manganese peroxidase [32]. In the case of *Pleurotus* fungi, the biodegradation process relies on the capacity of these mushrooms to produce the aforementioned enzymes, which exhibits their degrading properties against mycotoxins with varying efficiency depending on the type of toxins. According to the literature, AFB1 (with an in vitro degradation degree approximately 90–100%) [33,34,35] and ZEN (70–90% in vitro) [35,36] appear to be the most susceptible on the activity of *Pleurotus* spp. Other mycotoxins, including fumonisins, ochratoxin A, and deoxynivalenol, remain largely unexplored to this day, although some data suggest lower effectiveness against them compared to AFB1 and ZEN [36].

The aim of this study was to investigate the effectiveness of *Pleurotus ostreatus* in degrading AFB1 and ZEN present in the medium (a commercial substrate enriched with maize in 1:1 ratio) under in vivo conditions. This research represents the first in vivo study utilizing the mycelium of *P. ostreatus* for the degradation of these mycotoxins, specifically ZEN. Notably, until now, no in vivo experiments have been conducted to evaluate the degradation of ZEN, making this study a pioneering approach in exploring the potential of fungal mycelium for detoxification under such conditions.

## 2. Materials and Methods

### 2.1. Chemicals

Chemical standards AFB1 (5 mg) and ZEN (5 mg) were purchased in Romer Labs Division Holding GmbH. (Tulln an der Donau, Austria). AFB1 was dissolved in 5 mL of acetonitrile. HPLC-grade methanol and acetonitrile were purchased from Witko Sp. z o. o., (Łódź, Poland). Mycosep AflaZon columns were purchased from Romer Labs Division Holding GmbH. (Tulln an der Donau, Austria).

### 2.2. Cultivation of P. ostreatus in an AFB1- and ZEN-Contaminated Mushroom Medium

*Pleurotus ostreatus* was cultivated on the medium consisting of commercial substrate (Planto, Skierniewice, Poland) designed for production of mushrooms in home environment, with the addition of maize grains. The proportion of maize grain to the commercial substrate (sawdust) was 1:1. The maize grain was sterilized by autoclaving at 120 °C for 15 min to minimalize the risk of bacterial or fungal contamination. AFB1 or ZEN (concentration 1 mg/1 mL) was dissolved in hot water, which was then used to hydrate the substrate before the cultivation. After inoculation of medium with mycelium of *Pleurotus ostreatus*, the medium was divided into three equal portions for each toxin at each concentration level and transferred into 500 mL plastic containers. The cultivation process was carried out at 25 °C for 3 weeks, allowing the mycelium to fully colonize the medium. The next step involved applying the thermal shock by relocating the vessels with inoculated medium to specific conditions (15 °C, 80–90% humidity, and 12 hours of light per day). The mushrooms were maintained under these conditions for an additional 2 weeks until the first fruiting bodies became visible. The toxin concentration variants were selected as follows: AFB1 (100, 150, and 300 μg/kg, designated as Medium 1, 2, and 3, respectively) and ZEN (150, 300, and 1000 μg/kg, designated as Medium 4, 5, and 6, respectively). These concentration variants were chosen to assess the effectiveness of mushroom under challenging conditions.

### 2.3. Sample Preparation (AFB1 Measurement)

One gram of research material (lyophilized mushrooms or lyophilized medium) was weighed and homogenized in the Unidrive X 1000 homogenizer (CAT Scientific, Inc., Pase Robles, CA, USA) for 3 min with the addition of 10 mL of the mixture of acetonitrile and water (84:16 *v*/*v*). The samples were then centrifuged in the MPV-351R laboratory centrifuge (Med. Instruments, Warsaw, Poland) at 10,730× *g* for 10 min and filtered through a fine-grained filter. The extract (2.5 mL) was diluted in 10 mL of the mixture of acetonitrile and water (84:16 *v*/*v*), and 5 mL of this solution was applicated to a Mycosep 224 AflaZon column (Romer Labs). After purification, 1 mL of solution was evaporated in a vacuum evaporator (Heidolph Instruments, Schwabach, Germany). The resulting sediment was redissolved in 1 mL of the AFLA phase. Finally, 1 mL of the filtered solution was injected onto the chromatographic column via an autosampler.

### 2.4. Sample Preparation (ZEN Measurement)

The procedure began by weighing 1.25 g of the research material (lyophilized mushrooms or lyophilized medium) and adding 20 mL of the mixture of acetonitrile and water (80:20 *v*/*v*). The samples were homogenized in the Unidrive X 1000 homogenizer (CAT Scientific, Inc., Pase Robles, CA, USA) for 3 min. After homogenization, the samples were centrifuged in the MPV-351R laboratory centrifuge (Med. Instruments, Warsaw, Poland) at 10,730× *g* for 10 min and filtered through a fine-grained filter. The extract (4 mL) was applicated to Bond Elut Mycotoxin columns (Agilent Technologies Sp. z o. o, Warsaw, Poland) according to the attached procedure. After purification, 2 mL of solution was evaporated in a vacuum evaporator (Heidolph Instruments, Schwabach, Germany). The resulting sediment was redissolved in 0.5 mL of the 30% MeOH. Then, 0.5 mL of the filtered solution was injected onto the chromatographic column via an autosampler.

### 2.5. HPLC Analysis

The content of AFB1 in mushrooms and medium was determined by the Knauer K 1001 high-performance liquid chromatograph (KNAUER Wissenschaftliche Geräte GmbH, Berlin, Germany) coupled with the RF-10AXL (Shimadzu, Kyoto, Japan) fluorescence detector.

### 2.6. LC/MS Analysis

The content of ZEN in the mushroom and medium was determined using an H-class liquid chromatograph coupled to a mass spectrometer with a time-of-flight analyzer (UPLC-TOF-HRMS; Waters, Milford, MA, USA). Analytes were separated on a 2.1 × 100 mm, 1.6 µm UPLC C18 Cortecs chromatographic column (Waters) with an appropriate pre-column, operated with a gradient regime.

### 2.7. Method Validation

The verification of the developed analytical method was conducted by measurement of limit of quantification (LOQ, concentration of the given analyte at which its signal-to-noise ratio is 10:1), limit of detection (LOD, concentration at which the signal-to-noise ratio is 3:1), calibration curve linearity range, recovery rate R, and precision (repeatability expressed as relative standard deviation, RSD) for individual analytes. Generation of calibration curves for AFB1 was performed by preparing of 0.01 μg/mL (AFB1) standard solutions with the use of reference certified solutions of AFB1 in acetonitrile (Romer Labs, Tulln, Austria). Calibration curves were prepared in the concentration range of 1.25–20 μg/kg. The LOQ and LOD for both investigated analytes were 8.43 μg/kg and 2.53 μg/kg, respectively. In the case of ZEN, a matrix-matched template was used to verify the results. The matrix-matched template was prepared using the same method as real samples, but with the 1.25 g of matrix material free of ZEN (control sample—medium with mycelium without toxins). After the evaporation, 3.75, 7.5, 12.5, or 25 μL (the volume of standard was calculated according to the level of the concentration of ZEN in real samples; 150, 300, 1000 μg/kg) of the reference standard (5 μg/mL) was added, then the sediment was redissolved in 0.5 mL of the 30% MeOH. The LOQ and LOD for ZEN were 100 μg/kg and 30 μg/kg, respectively. Method repeatability and recovery rate were estimated using the results of analysis of three fortified samples for each of the investigated matrices (lyophilized mushrooms or lyophilized medium) for both toxins. Fortified samples were prepared by adding a reference solution of the respective toxin (AFB1 or ZEN) to the control matrix material, which consisted of the substrate with Pleurotus but without toxins. For AFB1, 1 g of matrix material was used, while for ZEN, 1.25 g of matrix material was used. The fortified samples were spiked at three concentration levels: 100, 150, and 300 μg/kg for AFB1 and 150, 300, and 1000 μg/kg for ZEN. The analysis of the samples was carried out using the same method as the real samples.

The European Commission (401/2006) established minimal criteria for assessment of analytical techniques used to determine mycotoxins, including AFB1 [37]. For analyte concentration ranges between 1 and 10 μg/kg, recovery rate (R) of the assessed method should fall within 80–120%, and precision (RSD) must not exceed 20%. For concentrations below 1 μg/kg, the corresponding criteria are 50–120% and not worse than 40% for precision.

### 2.8. Statistical Analysis

To determinate the significance of differences in the concentrations of AFB1 and ZEN in medium with and without mycelium of *P. ostreatus*, one-way analysis of variance (ANOVA) was performed. Prior to the analysis, the assumptions of normality and homogeneity of variances were verified using the Shapiro–Wilk test and Levene’s test, respectively. Post hoc comparisons were conducted using Fisher’s least significant difference (LSD) test to identify statistically significant differences (*p* < 0.05) between the control and the respective media. All statistical analyses were performed using Statistica software, version 14.0.

## 3. Results

### 3.1. The Results of Validation

The repeatability of the method for determining the content of mycotoxins AFB1 and ZEN, along with the recovery rate, was assessed based on the analysis of three fortified samples at each concentration level (Table 1).

A calibration curve was not developed for zearalenone. The results were processed using a pure standard (dissolved in MeOH) and a matrix standard to confirm or exclude the matrix effect. The obtained recoveries and RSD values (Table 1) are in accordance with the guidelines presented in Regulation No. 401/2006 concerning the reliability of analytical methods.

### 3.2. The Impact of Presence of AFB1 or ZEN in Medium on Mushroom Yield

Mushrooms were cultivated on medium with three levels of AFB1 concentration: Medium 1 (100 μg/kg), Medium 2 (150 μg/kg), and Medium 3 (300 μg/kg), as well as four levels of ZEN: Medium 4 (150 μg/kg), Medium 5 (300 μg/kg), and Medium 6 (1000 μg/kg). A control sample of pure medium (control sample) was also prepared to compare the fruiting body formation rate and yield size obtained from the medium contaminated with AFB1 or ZEN to those grown on a pure medium (without toxins). Macroscopic observations showed that the presence of both AFB1 and ZEN in the medium slowed down the mycelium development and delayed fruiting body formation; however, it did not ultimately negatively affect the yield size. Fruiting bodies from the control sample reached harvesting maturity around the 6th week of cultivation. In the case of the experimental samples (presence of AFB1 or ZEN), the harvest was delayed and typically occurred around the 7th–8th week of cultivation. However, the size and quality of the yield from the experimental samples (Medium 1, 2, 3, 4, 5, and 6 were at a similar level to the control sample (control sample). The results of the macroscopic analysis indicated that the presence of AFB1 or ZEN does not have a significant impact on the quality and yield size in the case of the *P. ostreatus* species.

### 3.3. The Estimation of AFB1 Concentration in Control Medium (Without Mycelium)

To estimate the degree of AFB1 degradation in a medium consisting of corn and sawdust in equal proportions (1:1), three concentration levels were chosen: 100, 150, and 300 μg/kg. Before analyzing the experimental samples, the toxin content in the control sample of the medium (without mycelium) was measured. The obtained results showed different AFB1 contents in the control samples at each of the three concentrations compared to the target values. For the control at the lowest concentration (100 μg/kg), the actual AFB1 content was 65.72 ± 1.95 μg/kg. For the next two concentration levels, the results were 89.4 ± 7.90 μg/kg (150 μg/kg) and 150.81 ± 4.07 μg/kg (300 μg/kg), respectively. The medium used was a non-homogeneous and complex matrix, which led to variations in the actual toxin levels compared to the target concentrations. These observations necessitated comparing the experimental sample results with the actual results of the control samples at the corresponding levels.

### 3.4. The Degradation of AFB1 by P. ostreatus in Contaminated Medium

A significant impact of *P. ostreatus* mycelium on the reduction in AFB1 content in the medium was observed at all concentration levels (*p* < 0.05, Table 2). The highest degree of degradation (87%) was achieved in Medium 1, with the lowest initial toxin concentration (100 μg/kg) in the medium. In this case, the average toxin content was approximately 8.32 ± 1.34 μg/kg, ranging from LOD to 21.58 μg/kg. The weakest reduction (53%) was observed in Medium 2 at the next concentration level (150 μg/kg), with AFB1 content ranging from 22.69 to 66.01 μg/kg, but even in this case, the observed reduction was statistically significant (*p* < 0.05). For Medium 3, at the highest initial concentration taken for the study (300 μg/kg), AFB1 content was reduced by 66%, with values ranging from LOQ to 91.97 μg/kg.

Fruiting bodies grown on toxin-contaminated medium at each selected concentration level were also analyzed. The AFB1 content in mature fruiting bodies was below the limit of quantification (LOQ) or the limit of detection (LOD) in most samples. AFB1 was detected in fruiting bodies grown on Medium 1 and Medium 2 at levels < LOD–LOQ. Only in the case of Medium 3 was AFB1 detected at a slightly higher level due to its presence in one sample, ranging from <LOD to 41.33 μg/kg.

Given these results, it was concluded that the fruiting bodies do not absorb or accumulate AFB1 in their cell walls, making them potentially safe for consumption by humans and animals. The statistical significance of these findings underscores the effectiveness of *P. ostreatus* mycelium in degrading AFB1, even at higher concentration levels. However, to support this conclusion, more studies are needed in this area, including a metabolomic analysis to determine whether AFB1 has been transformed into other derivative compounds by ligninolytic enzymes, which could be present in both the medium and the fruiting body cell walls.

### 3.5. The Estimation of ZEN Concentration in Control Medium (Without Mycelium)

Similarly to AFB1, ZEN levels were analyzed in the control medium sample (without mycelium). The target concentrations for the control samples were set at 150, 300, and 1000 μg/kg. However, the analysis results indicated that the actual ZEN concentrations in the control samples differed from the expected values. For the lower concentrations, 150 and 300 μg/kg, the actual values were 429 ± 138.17 and 684.3 ± 28.76 μg/kg, respectively. Regarding the higher concentrations, 1000 μg/kg, the result was 1400.28 ± 17.04 μg/kg. The medium, being a non-homogeneous and complex matrix, contributed to variations in the actual toxin levels compared to the target concentrations. This inherent variability made it challenging to achieve consistent distribution of ZEN in the samples. As a result, the final concentrations measured deviated from the expected values. The results of the experimental samples were compared to the actual concentrations present in the control medium samples.

### 3.6. The Degradation of ZEN by P. ostreatus in Contaminated Medium

The results of the analyses (Table 3) indicate that the mycelium of *P. ostreatus* significantly influences the degradation of ZEN in artificially contaminated medium (*p* < 0.05). A very high reduction was achieved at lower toxin concentration levels, reaching 97% for Medium 4 (150 μg/kg) and 99% for Medium 5. The ZEN content in Medium 4 and Medium 5 was below the detection limit (LOD = 30 μg/kg). For higher concentrations, in Medium 6 (1000 μg/kg), the degradation rate was 91%. The ZEN content in Medium 6 was present at an average level of 128.4 ± 11.58 μg/kg (min–max 61.8–189.7 μg/kg). ZEN was present in trace amounts in the mushroom fruiting bodies, demonstrating a significant reduction relative to the initial concentration (*p* < 0.05).

**Figure 1:**
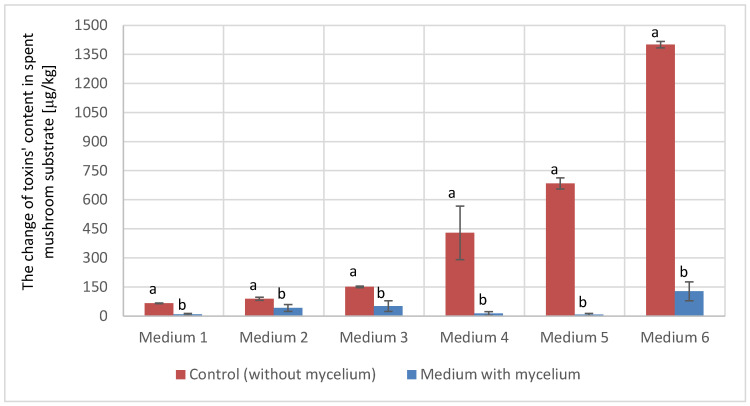
The reduction in toxins (AFB1 and ZEN) in spent mushroom medium after the cultivation. Medium 1 (100 μg/kg AFB1), Medium 2 (150 μg/kg AFB1), Medium 3 (300 μg/kg AFB1), Medium 4 (150 μg/kg ZEN), Medium 5 (300 μg/kg ZEN), and Medium 6 (1000 μg/kg ZEN). Groups labeled with different letters (a, b) indicate significant differences (*p* < 0.05) between the control and the respective medium based on one-way ANOVA.

Summarizing the obtained results (Figure 1), it can be observed that in the case of ZEN (with a reduction rate of 91–99% depending on the initial concentration), *P. ostreatus* mycelium had a more effective impact on toxin degradation compared to AFB1, where the reduction rate ranged from 53 to 87%.

## 4. Discussion

The literature data on the degradation of mycotoxins using fungi of the genus *Pleurotus* are limited due to the innovative nature of mycoremediation techniques, which have only gained significant popularity among researchers within the past decade. There are even fewer data on the effectiveness of this detoxification method in vivo, as most authors focus on the extraction of enzymes such as laccase and manganese peroxidase and evaluate their effectiveness against toxins in controlled in vitro conditions, sometimes employing mediators to catalyze this process. In the discussion, an effort was made to analyze the obtained results in comparison with those achieved in vivo by other authors and to present the current state of knowledge on the effectiveness of the method and the enzymes themselves in in vitro conditions.

The results obtained in our study indicate the effectiveness of *P. ostreatus* mycelium in degrading AFB1 at a level of 53–87%, depending on the toxin concentration. The potential accumulation of unchanged AFB1 in the cell walls of the fruiting bodies was ruled out; however, to estimate the safety for human or animal consumption, a comprehensive metabolomic analysis should be conducted in the future to detect the presence of any derivative compounds formed during degradation.

In the study by Brana et al. [28], cultivation of *Pleurotus eryngii* on artificially contaminated AFB1 medium consisting of a prepared substrate with 25% corn addition was conducted. After the cultivation period (approximately 42 days), an analysis of AFB1 content in the spent mushroom substrate (SMS) was performed. The results showed that AFB1 levels were reduced by 86%. These results are consistent with the findings of our study, and confirm the effectiveness of mycelium in neutralizing AFB1. The study also analyzed SMS and mushroom mats for the presence of AFB1 and aflatoxicol (AFOL), a less toxic derivative of AFB1, to rule out the possibility of accumulating these substances. Neither AFB1 nor AFOL were detected in the mushroom mats, which is consistent with the findings of our study, where the accumulation of AFB1 in the fruiting bodies was also ruled out.

Another study conducted by Jackson et al. [38] also demonstrated a high level of AFB1 reduction in the spent mushroom substrate (SMS), which consisted of a substrate with the addition of toxin-contaminated corn grains used for *P*. *ostreatus* cultivation, achieving a reduction rate of 94%. A slightly different study was conducted by Haidukowski et al. [39], who used lyophilized *P. eryngii* mycelium to examine the bioabsorption potential of mushrooms in the context of AFB1 removal. An 85% reduction in AFB1 content in the test samples was observed, indicating that, in addition to enzymatic activity, bioabsorption by the mycelium represents another mechanism of mycotoxin neutralization.

To date, no data have been available on the effectiveness of *Pleurotus* spp. mycelium against ZEN in in vivo conditions, making the experiment proposed in this study one of the first to explore this topic.

The degradation of mycotoxins using fungi from the genus *Pleurotus* has been more extensively described in vitro studies, where the focus has been on the effectiveness of enzymes extracted from the mycelium. Many studies have confirmed a high degree of AFB1 degradation using laccase produced by species such as *P. eryngii* [34,36] and *P. pulmonarius* [35,40]. Other authors have enhanced enzymatic activity by utilizing specific mediators to increase the efficiency of the degradation process [33,35,36,40,41]. In the study by Song et al. [35], it was confirmed that both AFB1 and ZEN could be completely degraded using laccase in the presence of the mediator ABTS (2,2′-azino-bis(3-ethylbenzothiazoline-6-sulfonic acid)) and an alkaline pH. Loi et al. [40] demonstrated the effectiveness of laccase against AFB1 and its derivative aflatoxin M1 (AFM1) in the presence of three redox mediators (SA-syringaldehyde, AS-acetosyringone, and ABTS). In another study, Loi et al. [36] tested the effectiveness of laccase against a broader spectrum of mycotoxins such as ZEN, deoxynivalenol (DON), fumonisin B1 (FB1), ochratoxin A (OTA), and T-2 toxin. The degree of degradation varied for each toxin and depended on the presence and selection of mediators (SA, ABTS, TEMPO-2,2,6,6-tetramethylpiperidin-1-yl, PhR: phenol red). ZEN was completely removed from the matrix, while FB1 and OTA were reduced by approximately 74% and 30%, respectively. Conversely, DON proved to be the most resistant toxin to the enzyme and enzyme–mediator complex.

The second enzyme with proven efficacy in mycotoxin removal is manganese peroxidase, which was the focus of studies by Yehia et al. [33] and Wang et al. [41]. Yehia et al. [33] indicated that after a 24 h incubation in the presence of the enzyme, the AFB1 level in the matrix decreased by 67%, and extending the incubation period to 48 h resulted in a 90% reduction in toxin concentration. Wang et al. [41] examined the degradation potential of manganese peroxidase isolated from other white rot fungi (WRF) such as *Phanerochaete chrysosporium, Irpex lacteus, Ceriporiopsis subvermispora,* and *Nematoloma frowardii* against AFB1, ZEN, DON, and FB1. The enzyme’s effectiveness was confirmed for these toxins, but only in the presence of the mediator malonic acid dicarboxylate. However, comparing the activity of both enzymes, laccase demonstrates higher effectiveness against mycotoxins (AFB1) compared to manganese peroxidase. Moreover, appropriate conditions such as temperatures ranging from 25 to 37 °C and pH ≥ 8 are most favorable for the degradation process [34].

Considering the data obtained from both in vitro and in vivo studies, mycoremediation appears to be a promising method for reducing mycotoxin levels in contaminated matrices. However, due to the relatively limited data on the use of this method in in vivo conditions and its effectiveness against other mycotoxins such as OTA, DON, and FB1, this research direction should be further expanded in the coming years.

The issue of metabolites formed during mycotoxin degradation and the evaluation of the toxicity of these substances also deserves the attention of researchers, particularly as the transformation of mycotoxins into various metabolites can significantly alter their toxicological profiles. Understanding the metabolic pathways of toxins like aflatoxin B1 (AFB1) and zearalenone (ZEN) is crucial for evaluating their potential risks to human and animal health. In particular, aflatoxin B1 (AFB1), the most toxic and carcinogenic representative of mycotoxins, is distinguished by its unique chemical structure. The complex structure of AFB1, comprising a cyclopentenone ring fused with a coumarin lactone system and a dihydrofurofuran moiety, makes it a compound with multiple reactive centers, which explains its high toxicity and the diversity of its degradation products [42]. The degradation of AFB1 leads to the formation of metabolites such as aflatoxicol (AFL) and aflatoxin M1 (AFM1), which, despite their reduced toxicity, still pose a threat to public health. Aflatoxicol (AFL), a product of the selective reduction of the carbonyl group in the cyclopentenone ring, exhibits significantly lower acute toxicity—up to 18 times lower than AFB1. Nevertheless, AFL retains its ability to induce carcinogenic effects, particularly in animal models such as fish and birds [43]. In turn, aflatoxin M1 (AFM1) is formed through the hydroxylation of AFB1 during liver metabolism. Although AFM1 exhibits only 10% of the mutagenic potential of its precursor form, it still retains carcinogenic and immunosuppressive properties, making it a significant concern for food safety [44]. In turn, the chemical structure of ZEN, consisting of a β-resorcylic acid lactone system linked to a 10-hydroxy-6-oxo-trans-1-undecenyl group, determines its biological activity and interactions with the endocrine system [45]. The main metabolites of ZEN metabolism are α-zearalenol (α-ZEL) and β-zearalenol (β-ZEL). Further metabolism of α-ZEL and β-ZEL leads to the formation of α-zearalanol (α-ZAL) and β-zearalanol (β-ZAL), with α-ZAL being predominantly converted into β-ZAL and, to a lesser extent, into zearalanone (ZAN). The degradation of ZEN and AFB1 by *Pleurotus* fungi may lead to the formation of products with unknown biological activity, which may exhibit lower, comparable, or even greater toxicological properties than the parent compound. Conducting a detailed metabolomic analysis will enable the identification and characterization of the degradation products of ZEN and AFB1 resulting from the activity of enzymes produced by Pleurotus. Metabolomics, as an advanced analytical tool, enables the generation of a complete metabolic profile and the determination of the chemical structure of the resulting products, which is crucial for assessing their potential risks to human and animal health. Understanding the degradation pathways of ZEN and AFB1 and their biological effects in the context of the ligninolytic enzyme activity of *Pleurotus* fungi is essential for a comprehensive risk assessment associated with mycoremediation techniques.

## 5. Conclusions

This study demonstrated the significant efficacy of *Pleurotus ostreatus* in degrading aflatoxin B1 (AFB1) and zearalenone (ZEN) in contaminated medium. The mycoremediation process, leveraging the ligninolytic enzymes produced by this white rot fungus, achieved reduction rates of 53–87% for AFB1 and 91–97% for ZEN, depending on the initial toxin concentrations. Notably, the mushrooms grown on contaminated medium contained negligible amounts of both toxins, suggesting minimal absorption and accumulation in the fruiting bodies, thus indicating their potential safety for consumption. These findings highlight the promise of *Pleurotus ostreatus* as an effective biotechnological tool for mycotoxin degradation, offering a safer and potentially more sustainable alternative to conventional chemical and physical detoxification methods. Further research, including comprehensive metabolomic analysis, is necessary to fully understand the transformation pathways of these toxins and to confirm the long-term safety and efficacy of this bioremediation approach.

## Figures and Tables

**Table 1:** Recovery rate and RSD results for the determination of AFB1 and ZEN.

	Level of Fortification (*n* = 3 × 3) [µg/kg]	Recovery Rate (%)	RSD (%)
AFB1	100	88–103	<20
	150	87–112	<20
	300	82–87	<20
ZEN	150	82–93	<20
	300	94–101	<20
	1000	89–108	<20

**Table 2:** The reduction in AFB1 in spent mushroom medium at three concentration levels (100, 150, and 300 μg/kg).

	Min–Max [µg/kg]	Average [µg/kg]	Median	Reduction [%]
Medium 1	LOD–21.58	8.32 ± 1.34 *	7.86	87
Medium 2	22.69–66.01	41.83 ± 4.06 *	40.83	53
Medium 3	LOQ–91.97	51.5 ± 10.84 *	48.46	66

Medium 1 (100 μg/kg), Medium 2 (150 μg/kg), Medium 3 (300 μg/kg), LOQ—8.43 μg/kg, LOD—2.53 μg/kg. * *p* < 0.05.

**Table 3:** The reduction in ZEN in spent mushroom medium at three concentration levels (150, 300, and 1000 μg/kg).

	Min–Max [µg/kg]	Average [µg/kg]	Median	Reduction [%]
Medium 4	<LOD	<LOD *	<LOD	97
Medium 5	<LOD	<LOD *	<LOD	99
Medium 6	61.8–189.7	128.4 ± 11.58 *	130.6	91

Medium 4 (150 μg/kg), Medium 5 (300 μg/kg), Medium 6 (1000 μg/kg), LOQ—100 μg/kg, LOD—30 μg/kg. * *p* < 0.05.

## Data Availability

The original contributions presented in this study are included in the article. Further inquiries can be directed to the corresponding author.

## References

[1] Alshannaq A., Yu J.H. (2017). Occurrence, Toxicity, and Analysis of Major Mycotoxins in Food. Int. J. Environ. Res. Public Health.

[2] Herrera M., Bervis N., Carramiñana J.J., Juan T., Herrera A., Ariño A., Lorán S. (2019). Occurrence and Exposure Assessment of Aflatoxins and Deoxynivalenol in Cereal-Based Baby Foods for Infants. Toxins.

[3] Ramadan A.N., Al-Ameri H.A. (2022). Aflatoxins.

[4] Warnatzsch E.A., Reay D.S., Camardo Leggieri M., Battilani P. (2020). Climate Change Impact on Aflatoxin Contamination Risk in Malawi's Maize Crops. Front. Sustain. Food Syst..

[5] Eskola M., Kos G., Elliott C.T., Hajšlová J., Mayar S., Krska R. (2019). Worldwide Contamination of Food-Crops with Mycotoxins: Validity of the Widely Cited ‘FAO Estimate’ of 25%. Crit. Rev. Food Sci. Nutr..

[6] Dieme R.M., Faye I., Zoclanclounon Y.A., Fonceka D., Ndoye O., Diédhiou P.M. (2018). Identification of Sources of Resistance for Peanut *Aspergillus flavus* Colonization and Aflatoxin Contamination. Int. J. Agron..

[7] Pickova D., Ostry V., Toman J., Malir F. (2021). Aflatoxins: History, Significant Milestones, Recent Data on Their Toxicity and Ways to Mitigation. Toxins.

[8] IARC Working Group on the Evaluation of Carcinogenic Risks to Humans, International Agency for Research on Cancer, World Health Organization (2002). Some Traditional Herbal Medicines, Some Mycotoxins, Naphthalene and Styrene.

[9] Gong Y.Y., Watson S., Routledge M.N. (2016). Aflatoxin Exposure and Associated Human Health Effects, a Review of Epidemiological Studies. Food Safety.

[10] Gheorghe-Irimia R.A., Tăpăloagă D., Tăpăloagă P.R., Ilie L.I., Șonea C., Serban A.I. (2022). Mycotoxins and Essential Oils—From a Meat Industry Hazard to a Possible Solution: A Brief Review. Foods.

[11] Negash D. (2018). A Review of Aflatoxin: Occurrence, Prevention, and Gaps in Both Food and Feed Safety. J. Appl. Microb. Res..

[12] Rushing B.R., Selim M.I. (2018). Aflatoxin B1: A Review on Metabolism, Toxicity, Occurrence in Food, Occupational Exposure, and Detoxification Methods. Food Chem. Toxicol..

[13] Singh U., Gupta S., Gupta M. (2021). A Review on Study on Biological Ill Effects and Health Hazards f Aflatoxins. Int. J. Adv. Med..

[14] Wogan G.N., Newberne P.M. (1967). Dose response characteristics of aflatoxin B, carcinogenesis in the rats. Cancer Res..

[15] Li H., Xing L., Zhang M., Wang J., Zheng N. (2018). The Toxic Effects of Aflatoxin B1 and Aflatoxin M1 on Kidney through Regulating L-Proline and Downstream Apoptosis. BioMed Res. Int..

[16] Sun Y., Song Y., Long M., Yang S. (2023). Immunotoxicity of Three Environmental Mycotoxins and Their Risks of Increasing Pathogen Infections. Toxins.

[17] Ropejko K., Twarużek M. (2021). Zearalenone and Its Metabolites—General Overview, Occurrence, and Toxicity. Toxins.

[18] Warth B., Preindl K., Manser P., Wick P., Marko D., Buerki-Thurnherr T. (2019). Transfer and Metabolism of the Xenoestrogen Zearalenone in Human Perfused Placenta. Environ. Health Perspect..

[19] Bulgaru C.V., Marin D.E., Pistol G.C., Taranu I. (2021). Zearalenone and the Immune Response. Toxins.

[20] Grgic D., Betschler A., Früholz R., Novak B., Varga E., Marko D. (2022). Estrogenic In Vitro Evaluation of Zearalenone and Its Phase I and II Metabolites in Combination with Soy Isoflavones. Arch. Toxicol..

[21] Mahato D.K., Pandhi S., Kamle M., Gupta A., Sharma B., Panda B.K., Srivastava S., Kumar M., Selvakumar R., Pandey A.K. (2022). Trichothecenes in Food and Feed: Occurrence, Impact on Human Health and Their Detection and Management Strategies. Toxicon.

[22] Gallo A., Mosconi M., Trevisi E., Santos R.R. (2022). Adverse Effects of Fusarium Toxins in Ruminants: A Review of In Vivo and In Vitro Studies. Dairy.

[23] Afshar P., Shokrzadeh M., Raeisi S.N., Ghorbani-HasanSaraei A., Nasiraii L.R. (2020). Aflatoxins Biodetoxification Strategies Based on Probiotic Bacteria. Toxicon.

[24] Akpasi S.O., Anekwe I.M.S., Tetteh E.K., Amune U.O., Shoyiga H.O., Mahlangu T.P., Kiambi S.L. (2023). Mycoremediation as a Potentially Promising Technology: Current Status and Prospects—A Review. Appl. Sci..

[25] Mohapatra D., Rath S.K., Mohapatra P.K., Prasad R. (2018). Bioremediation of Insecticides by White-Rot Fungi and Its Environmental Relevance. Mycoremediation and Environmental Sustainability.

[26] Bhattacharya S., Das A., Prashanthi K., Palaniswamy M., Angayarkanni J. (2013). Mycoremediation of Benzo[a]pyrene by *Pleurotus ostreatus* in the Presence of Heavy Metals and Mediators. 3 Biotech.

[27] Da Rocha Ferreira G.L., Vendruscolo F., Antoniosi Filho N.R. (2019). Biosorption of Hexavalent Chromium by *Pleurotus ostreatus*. Heliyon.

[28] Branà M.T., Cimmarusti M.T., Haidukowski M., Logrieco A.F., Altomare C. (2017). Bioremediation of Aflatoxin B1-Contaminated Maize by King Oyster Mushroom (*Pleurotus eryngii*). PLoS ONE.

[29] Kapahi M., Sachdeva S. (2017). Mycoremediation Potential of *Pleurotus* Species for Heavy Metals: A Review. Bioresour. Bioprocess..

[30] Chen L., Zhang X., Zhang M., Zhu Y., Zhuo R. (2022). Removal of Heavy-Metal Pollutants by White Rot Fungi: Mechanisms, Achievements, and Perspectives. J. Clean. Prod..

[31] Adebayo E.A., Elkanah F.A., Afolabi F.J., Ogundun O.S., Alabi T.F., Oduoye O.T. (2021). Molecular Characterization of Most Cultivated *Pleurotus* Species in Sub-Western Region Nigeria with Development of Cost-Effective Cultivation Protocol on Palm Oil Waste. Heliyon.

[32] Benavides V., Ciudad G., Pinto-Ibieta F., Robledo T., Rubilar O., Serrano A. (2024). Enhancing Laccase and Manganese Peroxidase Activity in White-Rot Fungi: The Role of Copper, Manganese, and Lignocellulosic Substrates. Agronomy.

[33] Yehia R.S. (2014). Aflatoxin Detoxification by Manganese Peroxidase Purified from *Pleurotus ostreatus*. Brazil. J. Microbiol..

[34] Brana M.T., Sergio L., Haidukowski M., Logrieco A.F., Altomare C. (2020). Degradation of Aflatoxin B1 by a Sustainable Enzymatic Extract from Spent Mushroom Substrate of *Pleurotus eryngii*. Toxins.

[35] Song Y., Wang Y., Guo Y., Qiao Y., Ma Q., Ji C., Zhao L. (2021). Degradation of Zearalenone and Aflatoxin B1 by Lac2 from *Pleurotus pulmonarius* in the Presence of Mediators. Toxicon.

[36] Loi M., Fanelli F., Cimmarusti M.T., Mirabelli V., Haidukowski M., Logrieco A.F., Caliandro R., Mule G. (2018). In Vitro Single and Combined Mycotoxins Degradation by Ery4 Laccase from *Pleurotus eryngii* and Redox Mediators. Food Control.

[37] European Commission (2014). Commission Regulation (EC) No 519/2014 of 16 May 2014 amending Regulation (EC) No 401/2006 as regards methods of sampling of large lots, spices and food supplements, performance criteria for T-2, HT-2 toxin and citrinin and screening methods of analysis. Off. J. Eur. Union.

[38] Jackson L.W., Pryor B.M. (2017). Degradation of Aflatoxin B1 from Naturally Contaminated Maize Using the Edible Fungus *Pleurotus ostreatus*. AMB Express.

[39] Haidukowski M., Casamassima E., Cimmarusti M.T., Branà M.T., Longobardi F., Acquafredda P., Logrieco A., Altomare C. (2019). Aflatoxin B1-Adsorbing Capability of *Pleurotus eryngii* Mycelium: Efficiency and Modeling of the Process. Front. Microbiol..

[40] Loi M., Fanelli F., Zucca P., Liuzzi V.C., Quintieri L., Cimmarusti M.T., Monaci L., Haidukowski M., Logrieco A.F., Sanjust E. (2016). Aflatoxin B_1_ and M_1_ Degradation by Lac2 from *Pleurotus pulmonarius* and Redox Mediators. Toxins.

[41] Wang L., Huang W., Shen Y., Zhao Y., Wu D., Yin H., Wang J. (2022). Enhancing the Degradation of Aflatoxin B1 by Co-Cultivation of Two Fungi Strains with the Improved Production of Detoxifying Enzymes. Food Chem..

[42] Janik E., Niemcewicz M., Ceremuga M., Stela M., Saluk-Bijak J., Siadkowski A., Bijak M. (2020). Molecular Aspects of Mycotoxins—A Serious Problem for Human Health. Int. J. Mol. Sci..

[43] Karabulut S., Paytakov G., Leszczynski J. (2014). Reduction of Aflatoxin B1 to Aflatoxicol: A Comprehensive DFT Study Provides Clues to Its Toxicity. J. Sci. Food Agric..

[44] Marchese S., Polo A., Ariano A., Velotto S., Costantini S., Severino L. (2018). Aflatoxin B1 and M1: Biological Properties and Their Involvement in Cancer Development. Toxins.

[45] Mahato D.K., Devi S., Pandhi S., Sharma B., Maurya K.K., Mishra S., Dhawan K., Selvakumar R., Kamle M., Mishra A.K. (2021). Occurrence, Impact on Agriculture, Human Health, and Management Strategies of Zearalenone in Food and Feed: A Review. Toxins.

